# T lymphocyte subset imbalances in patients contribute to ankylosing spondylitis

**DOI:** 10.3892/etm.2014.2046

**Published:** 2014-11-04

**Authors:** CHENGGONG WANG, QIANDE LIAO, YIHE HU, DA ZHONG

**Affiliations:** Department of Orthopedics, Xiangya Hospital, Central South University, Changsha, Hunan 410008, P.R. China

**Keywords:** ankylosing spondylitis, T lymphocyte subsets, cytokines, imbalance, inflammation

## Abstract

Ankylosing spondylitis is a chronic inflammatory rheumatic disease, which is characterized by inflammation of the spine and the sacroiliac joints. To date, the disease etiology remains unclear. In the present study, the correlation of T lymphocyte subset changes with the progression of ankylosing spondylitis was investigated. A total of 55 patients with ankylosing spondylitis (22 severe and 23 mild cases) and 20 healthy individuals were selected. Firstly, the punctured cells in the lesions and the serum were collected, and the lymphocytes and the peripheral blood mononuclear cells were prepared. Secondly, quantitative PCR, ELISA and flow cytometry analyses were carried out to detect the levels of a series of immunoglobulins, complements, helper T cells, cytotoxic T cells, regulatory cells and cytokines. The expression levels of α-globulin, γ-globulin, immunoglobulin (Ig)G, IgA, IgM, serum complement C3, and complement C4 were found to be significantly increased in ankylosing spondylitis patients. In addition, the percentage of Th1 and Th17 cells was found to be significantly higher in the ankylosing spondylitis groups (mild and severe) compared with the healthy individuals. As a result, the Th1/Th2 and Th17/Treg ratios were significantly higher in patients with ankylosing spondylitis. In addition, T lymphocyte subset ratio imbalances contributed to an increased expression of immune mediators, including interferon (IFN)-γ and interleukin (IL)-17A. The mRNA and protein expression levels of IFN-γ and IL-17A were found to be higher in the ankylosing spondylitis groups compared with the control group. The present study provided further evidence on the function and underlying mechanism of T lymphocyte subsets, which may be useful in the diagnosis and treatment of ankylosing spondylitis.

## Introduction

Ankylosing spondylitis, a chronic inflammatory rheumatic disease, is characterized by inflammation of the spine and the sacroiliac joints, which may induce new bone formation in the affected areas (1,2). The development of spinal syndesmophytes, resulting in ankylosis between vertebral bodies, is a common symptom of the disease. Upon ankylosis of the vertebral bodies, the entire spine is distorted and may form a ‘bamboo spine’ (3). Ankylosing spondylitis-induced structural damage triggers further damage involving changes in the physical pathology and mobility of the spine. Clinical therapy and diagnosis is mainly based on the radiographic progression of ankylosing spondylitis (4). Radiographic imaging is the most popular and convenient method used in the diagnosis and prognosis estimation of the disease. However, only severe ankylosing spondylitis-induced spinal damage can be identified through radiographic detection (5). Thus, understanding the molecular progression of ankylosing spondylitis would aid early diagnosis and treatment during pathogenesis.

To date, the cause of the disease remains unclear. The two main characteristics of ankylosing spondylitis are inflammation and bone reformation (6,7). Previous studies have demonstrated that inflammation is induced by bone reformation (8). In addition, several studies have indicated that genetic influence of the HLA-B27 gene is responsible for ankylosing spondylitis (9,10). HLA-B27 and bacterial infection have been considered to play a crucial role in the pathogenesis of spondyloarthritis (11). This viewpoint was supported by the findings of studies on *Chlamydia trachomatis*, *Shigella*, *Salmonella*, *Yersinia* and *Campylobacter,* which were shown to result in spondyloarthritis (12). Furthermore, T cell have been found to respond to aggrecan in ankylosing spondylitis (13), indicating that T cells plays a key role in the pathogenesis of ankylosing spondylitis. Previous studies demonstrated that the T cell subtypes, CD4^+^ and CD8^+^, may be triggered by aggrecan in the blood and synovial fluid specimens (13,14). In addition, tumour necrosis factor-α (TNF-α), an indicator of inflammation, has been found to be highly expressed in the sacroiliac joints, indicating that ankylosing spondylitis progression may be associated with the degree of inflammation (15). While the pathogenic cause of ankylosing spondylitis remains unclear, previous studies have revealed a T cell response to aggrecan, which provides valuable information on the disease pathogenesis.

T lymphocytes are considered to be crucial cells in the regulation of the immune system (16). Based on the receptors on T lymphocyte membranes, the cells are divided into a number of subtypes, including CD4^+^ and CD8^+^ cells. CD4^+^ cells are divided further into T helper (Th)1, Th2, Th17 and regulatory T (Treg) subsets, while CD8^+^ cells are divided into T cytotoxic (Tc)1, Tc2 and Tc17 subsets (17). Furthermore, Th1 and Tc1 cells secrete type 1 cytokines, including interleukin (IL)-2, TNF-α and interferon (IFN)-γ, while Th2 and Tc2 cells secrete type 2 cytokines, including IL-4, IL-5 and IL-13 (18). Other cytokines, including IL-10 and transforming growth factor (TGF)-β, are secreted by Treg cells, while IL-17 is secreted by Th17 and Tc17 cells (19,20). The specific secretion of different T lymphocytes cells is balanced in healthy individuals.

The aim of the present study was to: (i) Understand the balance changes in the secretion of T lymphocyte subtypes in ankylosing spondylitis patients; and (ii) illustrate the expression level changes of inflammation mediators, including IFN-γ, IL-17A, IL-4 and TGF-β, in ankylosing spondylitis patients. The study results provide evidence for future diagnostic and therapeutic strategies of ankylosing spondylitis.

## Materials and methods

### Clinical samples and preparation

All the clinical samples were obtained from the Department of Orthopaedics, Xiangya Hospital of Central South University (Changsha, China). In total, 55 patients, confirmed (via physical, X-ray and blood examinations) to suffer from ankylosing spondylitis, participated in the study from June 2011 to July 2013 and the disease severity was diagnosed according to the guidelines of the German Spondyloarthritis Inception Cohort (Table I) (Rudwaleit). Spinal radiographs (Multix Select DR; Siemens AG Healthcare, Erlangen, Germany), were obtained from the 55 ankylosing spondylitis patients. In addition, 20 healthy individuals with no symptoms of ankylosing spondylitis were enrolled into the study as the control group. Informed written consent was obtained from all patients/patients’ families and healthy individuals prior to participation in the present study. The clinical sample collection was approved by the Ethics Committee of the Xiangya Hospital of Central South University.

Punctured cells were collected from the patient lesions and stored at −80°C prior to the assay. A total of 5 ml serum was obtained from each patient which was subsequently centrifuged at 724 × g for 10min and 10–25°C (Eppendorf 5415C; Eppendorf, Hamburg, Germany). The lymphocytes were prepared using a lymphocyte kit (Lymphoprep^™^ Axis-Shield PoC AS, Oslo, Norway). The peripheral blood mononuclear cells were prepared in RPMI 1640 containing 10% fetal bovine serum (Gibco Life Technologies, Grand Island, NY, USA).

### Quantitative polymerase chain reaction (PCR)

Gene expression levels were detected using quantitative PCR. The samples were homogenized and total RNAs were isolated using TRIzol reagent (Invitrogen Life Technologies, Carlsbad, CA, USA). Gene expression level detection was performed using the 7500 Real-Time PCR System (Applied Biosystems Life Technologies, Foster City, CA, USA). The PCR conditions were as follows: DNA degeneration at 95°C for 5 sec, followed by primer annealing at 60°C for 40 sec and primer extension at 70°C for 90 sec. These steps were repeated for 40 cycles. The gene expression levels were calculated using the SDS software 1.3 (Applied Biosystems, Grand Island, NY, USA) of the PCR device. Melting-curve analysis was performed to confirm the specificity of the amplification products.

### Enzyme-linked immunosorbent assay (ELISA)

The serum levels of IFN-γ, IL-4, TGF-α (all Quantikine; R&D Systems, Inc., Minneapolis, MN, USA) and IL-17A (Heterodimer DuoSet, 5 Plate; R&D Systems, Inc.) were detected using ELISA kits according to the manufacturer’s instructions. The expression levels of the immune factors were assayed in triplicate to improve accuracy.

### Flow cytometric analysis

T lymphocyte subsets were analyzed by flow cytometry (MoFlo^®^ Astrios^™^; Beckman Coulter, Fullerton, CA, USA). The cells were stained using PerCP-Cy5.5-labeled anti-human CD3 (Anti-Human CD3 PerCP-Cyanine5.5; eBioscience, San Diego, CA, USA) and fluorescein isothiocyanate (FITC)-labeled anti-human CD8 (Anti-Human CD8a; eBioscience). Treg cells were identified using FITC-labeled anti-human CD4 (eBioscience). The various T lymphocyte subsets were calculated using the antibody signals of the specific proteins.

### Statistical analysis

The data were statistically analyzed using the SPSS version 17.0 software (SPSS, Inc., Chicago, IL, USA) and are displayed as the mean ± standard error of mean. Analysis of the significant differences among the study groups was performed by one-way analysis of variance. P≤0.05 was considered to indicate a statistically significant difference.

## Results

### Case summary

In total, 23 patients were found to suffer from mild ankylosing spondylitis (including normal or narrow joint clearance, no ‘bamboo spine’ and a bone dense region present), whereas 22 patients were found to suffer from severe ankylosing spondylitis (including no joint clearance, a ‘bamboo spine’ and no bone dense region present). As shown in Table I, no statistically significant differences were observed in the age, gender and duration of the disease between the two ankylosing spondylitis groups. The expression levels of α-globulin, γ-globulin, immunoglobulin (Ig)G, IgA, IgM, serum complement C3 and C4 were found to be significantly increased in ankylosing spondylitis patients. In addition, the expression levels of these indexes were found to be higher in severe ankylosing spondylitis patients compared with mild ankylosing spondylitis patients (Table I).

**Figure 1:**
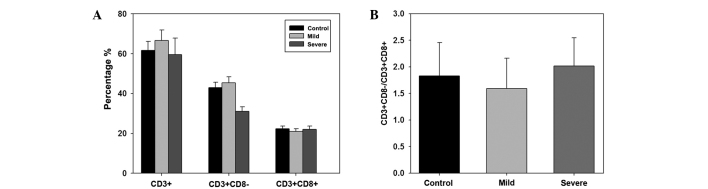
Changes in (A) the percentage of CD3^+^, CD3^+^CD8^−^ and CD3^+^CD8^+^ cells and (B) the CD3^+^CD8^−^/CD3^+^CD8^+^ ratio among the control, mild ankylosing spondylitis and severe ankylosing spondylitis groups.

The T lymphocyte subset content was analyzed in the control, mild ankylosing spondylitis and severe ankylosing spondylitis groups. In total, in the control group, the percentage of CD3^+^, CD3^+^CD8^−^ and CD3^+^CD8^+^ T lymphocyte subset cells were 61.60±4.61, 43.01±2.63 and 22.31±1.36%, respectively. In the mild ankylosing spondylitis group, the percentage of CD3^+^, CD3^+^CD8^−^ and CD3^+^CD8^+^ T lymphocyte subset cells were 66.62±5.32, 45.32±3.01 and 21.06±1.30%, respectively. In the severe ankylosing spondylitis group, the percentage of CD3^+^, CD3^+^CD8^−^ and CD3^+^CD8^+^ T lymphocyte subset cells were 59.61±8.20, 31.03±2.30 and 22.03±1.69%, respectively. However, no statistically significant differences were observed among the groups (Fig. 1A). In addition, the CD3^+^CD8^−^/CD3^+^CD8^+^ ratio in each group was calculated. The results indicated no statistically significant differences among the ratios of the three groups (Fig. 1B).

### Imbalance of Th1/Th2 and Tc1/Tc2 in ankylosing spondylitis patients

**Figure 2:**
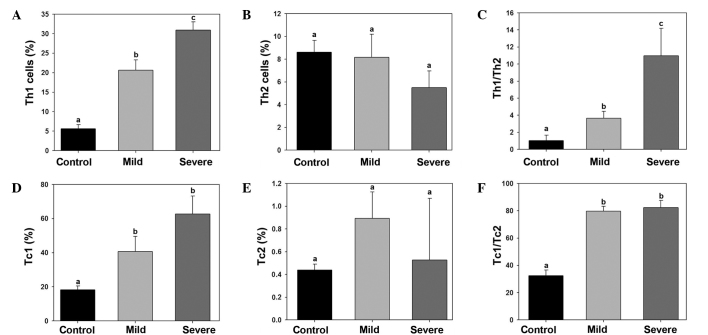
Th1/Th2 and Tc1/Tc2 ratio imbalance in patients with ankylosing spondylitis. The percentages of (A) Th1 and (B) Th2 cells, (C) Th1/Th2 ratio, percentages of (D) Tc1 and (E) Tc2 cells and (F) Tc1/Tc2 ratio are shown for the control, mild ankylosing spondylitis and severe ankylosing spondylitis groups. Different letters indicate a significant difference among the groups (P<0.05). Th, T helper; Tc, T cytotoxic.

The percentage of Th1, Th2, Tc1 and Tc2 cells in the control, mild ankylosing spondylitis and severe ankylosing spondylitis samples were calculated. The Th1 cell percentage was significantly increased in the mild and severe ankylosing spondylitis group (Fig. 2A). By contrast, no statistically significant differences were observed in the percentage of Th2 cells among the groups. Thus, the Th1/Th2 ratio was significantly higher in the mild and severe ankylosing spondylitis groups (Fig. 2C). The number of Tc1 cells was found to be significantly higher in the mild and severe ankylosing spondylitis groups compared with the control group (Fig. 2D). However, no statistically significant differences were observed in the percentage of Tc2 cells among the groups. Therefore, the Tc1/Tc2 ratio was significantly higher in the mild and severe ankylosing spondylitis groups compared with the control group (Fig. 2F).

### Imbalance of Th17/Treg in ankylosing spondylitis patients

**Figure 3:**
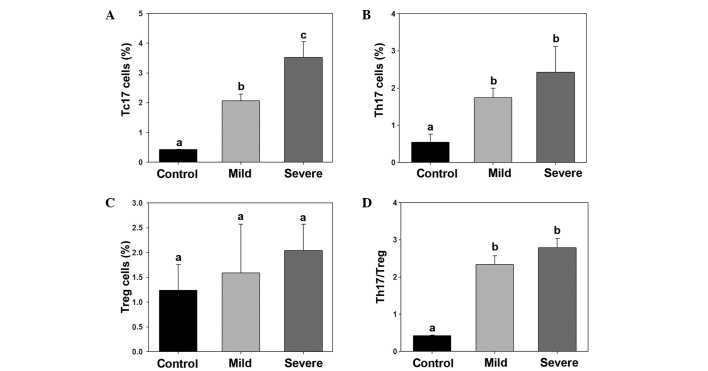
Percentages of (A) Tc17, (B) Th17 and (C) Treg cells, as well as (D) the Th17/Treg ratio, are shown for the control, mild ankylosing spondylitis and severe ankylosing spondylitis groups. Different letters indicate a significant difference among the groups (P<0.05). Tc, T cytotoxic; Th, T helper; Treg, regulatory T.

The percentage of Tc17, Th17 and Treg cells was calculated in the control, mild ankylosing spondylitis and severe ankylosing spondylitis groups. The percentage of Tc17 and Th17 cells were significantly increased in the mild and severe ankylosing spondylitis groups, whereas the percentage of Treg cells was not found to be significantly different among the groups. Thus, the Th17/Treg ratio was found to be significantly higher in the mild and severe ankylosing spondylitis groups (Fig. 3).

### Cytokine expression level changes in ankylosing spondylitis patients

The expression levels of IFN-γ, IL-17A, IL-4 and TGF-β were detected in the lesion punctured cells and the serum using quantitative PCR and ELISA, respectively. The mRNA expression levels in punctured cells and the serum protein expression levels of IFN-γ and IL-17A were found to be significantly higher in the mild and severe ankylosing spondylitis groups compared with the control group. In addition, the mRNA expression levels of IL-4 and TGF-β were found to be higher in punctured cells of the mild and severe ankylosing spondylitis groups. By contrast, no statistically significant differences were observed in the serum protein expression levels of these cytokines among the three groups.

## Discussion

A number of studies have indicated that certain pathogenic inflammation is associated with the development of ankylosing spondylitis (21,22). Although understanding the inflammatory mechanism of the disease progression is essential, the adaptive immunity of ankylosing spondylitis in patients has been rarely studied. The results of the present study demonstrate the variations in T lymphocyte subsets in mild and severe ankylosing spondylitis patients. From the results of this study, there does not appear to be an association between the number of T lymphocyte subset cells (CD3+, CD3+CD8− and CD3+CD8+) and the occurrence or severity of ankylosing spondylitis. Th1, Th17, Tc1 and Tc17 cell percentages were found to be significant higher in ankylosing spondylitis patients compared with the control group. In addition, the specific expression of immune mediators, such as IFN-γ and IL-17A, was found to be significantly increased in the plasma of ankylosing spondylitis patients compared with the control group. The results indicate that the progression of ankylosing spondylitis is associated with the imbalance of T lymphocyte subset. In 2009, Lin *et al* investigated the imbalance of blood B-cell subsets in ankylosing spondylitis patients (23). The imbalance of serum B-cell subsets was found to promote the development of ankylosing spondylitis and result in joint proliferation. Therefore, the increasing number of CD19^+^ cells was considered to be responsible for the progression of ankylosing spondylitis. Wu *et al* identified Th/Treg cell imbalance in ankylosing spondylitis patients and hypothesized that immunomodulation may contribute to the pathogenesis of ankylosing spondylitis through Th/Treg cell misbalance (24). Therefore, the T lymphocyte subset imbalance is a key factor in the incidence of ankylosing spondylitis pathogenesis.

In 2003, Kidd hypothesized that Th1/Th2 is associated with human health and disease progression development (25). Th1 and Th2 cells participate in various pathways of the immune system. Th1 cells drive the cellular immunity, namely the type-1 pathway, while Th2 cells drive the humoral immunity, namely the type-2 pathway. Th1 cells play a crucial role in disease pathogenesis and can cause cell death in tumors, while Th2 cells have been shown to upregulate antibody production. Changes in the Th1/Th2 ratio may alter the immunological balance, which is a suitable model for a number of diseases, including diabetes, cancer and pathogen infection (26–29). However, the Th1/Th2 ratio is not suitable for certain diseases, such as rheumatoid arthritis or asthma (30). In the present study, the Th1/Th2 ratio was significantly increased in patients with ankylosing spondylitis. In addition, the ratio was higher in severe ankylosing spondylitis patients compared with mild ankylosing spondylitis patients. Therefore, the Th1/Th2 balance and Th17/Treg balance were disturbed during the disease. A number of studies have reported that the Treg and Th17 subsets are associated with multidirectional immunology, which is in accordance with the results of the current study (31–33). Thus, Th1/Th2 and Th17/Treg imbalances may be due to the development of ankylosing spondylitis, indicating that these subsets may be crucial in the progression of the disease, particularly in severe ankylosing spondylitis. In the present study, the percentage of Th17 cells was found to increase in patients with ankylosing spondylitis. Subsequently, the specific expression of IL-17A (expressed by Th17 cells) was significantly stimulated. Considering that IL-17A plays an important role in the inflammatory response induced by neutrophil activation, the upregulation of Th17 cells in patients with ankylosing spondylitis is a crucial inflammatory pathway (34). The observations in the present study indicate that changes in the Th1/Th2 and Th17/Treg ratios are key evidence for the suffering of ankylosing spondylitis. Furthermore, the percentage of Th17 cells and expression level of IL-17A were found to be significantly higher in ankylosing spondylitis patients, while the protein levels of IL-4 and TGF-β were unchanged. The percentage of Treg cells was also unchanged in ankylosing spondylitis patients compared with the control group, indicating that Treg cells may not participate in the disease progression, as opposed to Th17 cells.

In the present study, the protein levels of IL-4 and TGF-β were found to be unchanged in ankylosing spondylitis patients (mild and severe) compared with the control group, which is in accordance with a previous study (35). Therefore, IL-4 and TGF-β may not participate in the progression of ankylosing spondylitis. Further studies are required to fully investigate the effect and underlying mechanism of T lymphocyte subsets in patients with ankylosing spondylitis.

In conclusion, imbalances in the T lymphocyte subset ratios, Th1/Th2 and Th17/Treg, were demonstrated in patients with ankylosing spondylitis. These imbalances resulted in increased mRNA and protein expression levels of immune mediators, particularly IFN-γ and IL-17A. The present study provided further evidence on the function and underlying mechanism of T lymphocyte subsets, which may be useful in the diagnosis and treatment of ankylosing spondylitis.

## Figures and Tables

**Figure 4:**
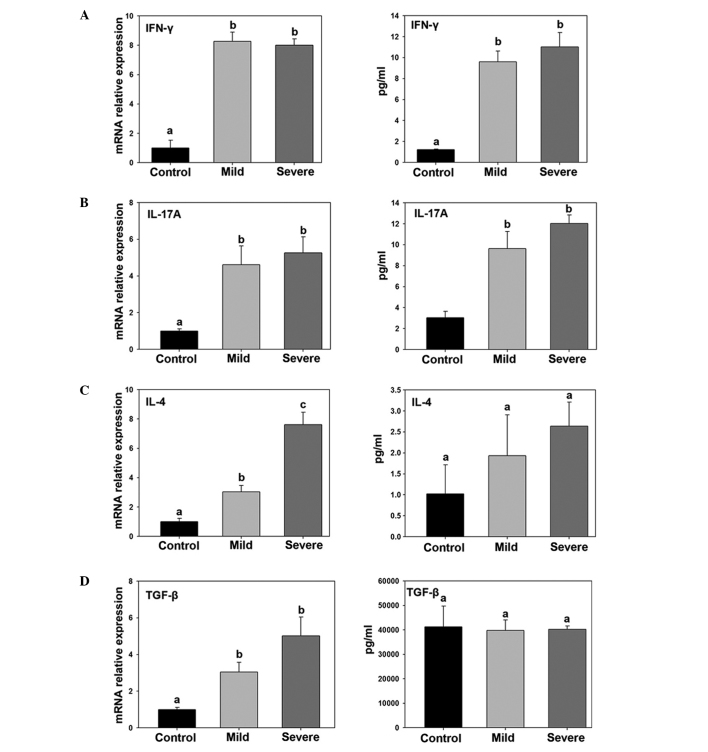
Relative mRNA and protein expression levels of (A) IFN-γ, (B) IL-17A, (C) IL-4 and (D) TGF-β are shown for the control, mild ankylosing spondylitis and severe ankylosing spondylitis groups. Different letters indicate a significant difference among the groups (P<0.05). IFN, interferon; IL, interleukin; TGF, tumor growth factor.

**Table I:** Summary of study group characteristics and observed expressions.

Variable	Control group	Mild ankylosing spondylitis group	Severe ankylosing spondylitis group
Number of samples (n)	20	23	22
Age (years)	55.05±6.42	52.31±8.24	51.22±6.78
Gender, male/female (n)	10/10	12/11	11/11
Duration of disease (years)	-	2.7±2.56	3.4±2.45
α-globulin (g/cl)	0.22±0.05	0.52±0.12^a^	0.68±0.08^a^
γ-globulin (g/cl)	0.37±0.12	0.98±0.29^a^	0.78±0.11^a^
IgG (mg/cl)	827.23±121.69	1328±48.90^a^	1438±104.87^a^
IgA (mg/cl)	12.55±1.95	21.69±2.62^a^	29.62±3.58^a^
IgM (mg/cl)	10.64±2.69	25.62±1.29^a^	31.57±2.02^a^
Serum complement C3 (U/ml)	88.65±10.62	152.70±26.62^a^	180.59±20.55^a^
Serum complement C4 (U/ml)	70.52±20.64	198.85±38.25^a^	201.92±44.52^a^

a P<0.05, vs. control group.

Data are presented as the mean ± standard error of mean. Ig, immunoglobulin.
